# Supplementary material for: Enhanced Insecticidal Effect and Interface Behavior of Nicotine Hydrochloride Solution by a Vesicle Surfactant
Source: Molecules. 2022 Oct 15;27(20):6916. doi: 10.3390/molecules27206916 (PMC9608593; doi:10.3390/molecules27206916)
Supplement: Supplementary file 1 [file molecules-27-06916-s001.zip › molecules-1927283-supplementary.pdf]

Supporting Information

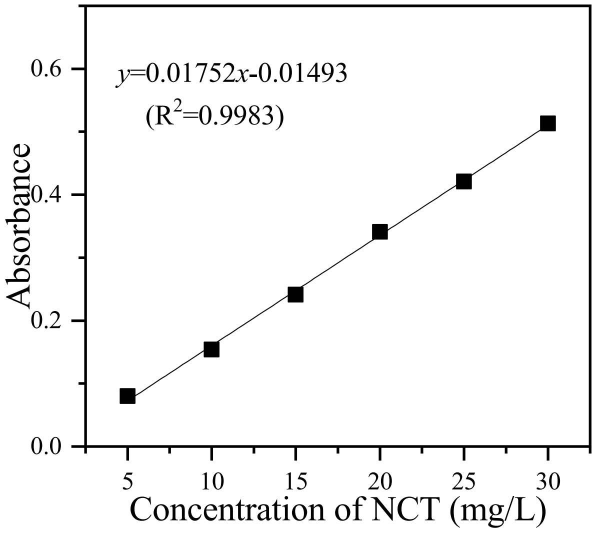

Figure S1. Standard curve of NCT/water solution.

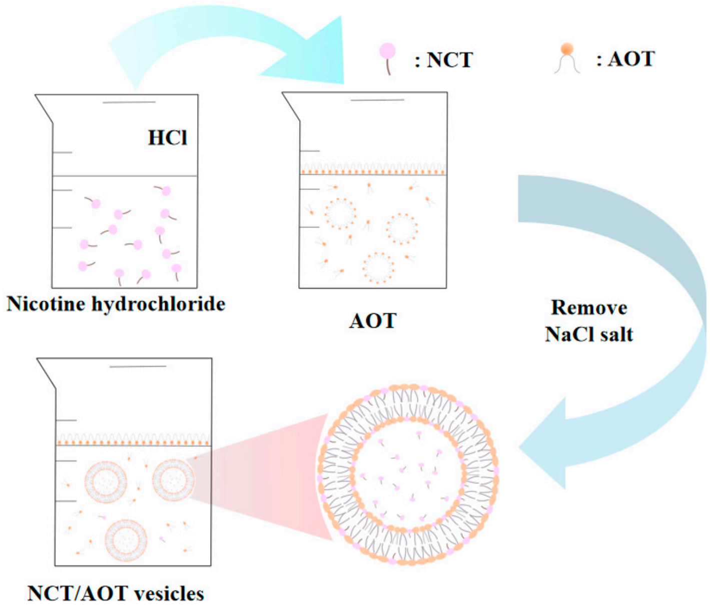

Scheme S1. The preparation process of NCT/AOT vesicles.

Table S1. Toxicity of NCT/AOT to Aphids.

Sample	[a]LC_{50}/ppm	Regression Equation	Correlation Coefficient
NCT	91.68	Y=0.478X+4.063	0.99
AOT	99.03	Y=4.271X-3.524	0.94
NCT/AOT	10.55	Y=2.370X+2.575	0.99

[a] Lethal concentration that cause 50% mortality.

Table S2. pH of NCT/AOT at different molar ratio of NCT and (AOT+NCT).

**Molar Ratio**	1/10	2/10	3/10	4/10	5/10	6/10	7/10	8/10	9/10
**pH**	2.23	1.73	1.46	1.31	1.29	1.12	1.04	0.98	0.92

Table S3. Particle size, PDI and Zeta potential of NCT/AOT at different dilution ratio.

**Dilution Ratio**	0	200	500	1000	2000
**Particle size (nm)**	198±2	187±2	186±3	178±1	159±1
**PDI**	0.23	0.21	0.23	0.15	0.10
**Zeta potential (mV)**	-56.5±1.1	-54.8±2.2	-43.6±0.3	-39. 0±0.8	-32.8±2.0
